# Metronidazole’s hidden impact: decoding cerebellar clues on MRI through a case report

**DOI:** 10.1093/omcr/omaf024

**Published:** 2025-05-28

**Authors:** Basma Beqqali, Joud Boutaleb, Basma Dghoughi, Lina Lasri, I Z I Zineb, Mohamed Bouchentouf, Tarik Salaheddine, Rachida Saouab, Meryem Edderai, Jamal Elfenni

**Affiliations:** Radiology department, Mohammed V military hospital, Rabat 10100, Morocco; Radiology department, Mohammed V military hospital, Rabat 10100, Morocco; Radiology department, Mohammed V military hospital, Rabat 10100, Morocco; Radiology department, Mohammed V military hospital, Rabat 10100, Morocco; Radiology department, Mohammed V military hospital, Rabat 10100, Morocco; Mohammed V military hospital, Rabat 10100, Morocco; Radiology department, Mohammed V military hospital, Rabat 10100, Morocco; Radiology department, Mohammed V military hospital, Rabat 10100, Morocco; Radiology department, Mohammed V military hospital, Rabat 10100, Morocco; Radiology department, Mohammed V military hospital, Rabat 10100, Morocco

**Keywords:** metronidazole, neurotoxicity, encephalopathy, drug-induced lesions

## Abstract

A 70-year-old patient with a history of skull base osteomyelitis experienced slurred speech, dizziness, and coordination issues. He had been treated with both intravenous and oral metronidazole. Brain MRI revealed symmetrical areas of hyperintensity in the bilateral dentate nuclei, tectum, and splenium of the corpus callosum. These abnormalities nearly completely disappeared a few days after stopping the medication. Metronidazole, frequently used as an antiparasitic and antibacterial drug, can lead to neurotoxicity, particularly when used for extended periods. Symptoms generally improve after the drug is discontinued. MRI is vital for diagnosis and follow-up, as it detects specific changes in brain regions due to axonal swelling and increased water content.

## Introduction

Metronidazole is a widely used antiparasitic and antibacterial medication, commonly prescribed for various infections. While generally well-tolerated, prolonged use or high doses can lead to rare but significant neurotoxic effects, including cerebellar toxicity. MRI plays a crucial role in diagnosing and monitoring metronidazole-induced neurotoxicity, revealing characteristic changes in specific brain regions

## Case report

A 70-year-old male patient, with a medical history of type 2 diabetes and myasthenia gravis, and no toxic habits, presented with a sudden onset of vertigo, ataxia, and slurred speech that had been present for two days. The patient was a known case of skull base osteomyelitis caused by *Pseudomonas aeruginosa*. He had initially been treated with intravenous metronidazole at a dose of 1500 mg per day for five days, followed by oral metronidazole for five weeks.

Approximately six weeks after starting treatment, with a cumulative dose of 60 g, he presented to our hospital with cerebellar symptoms as described above. Clinical examination revealed positive cerebellar signs, including dysdiadochokinesia, a positive Romberg’s sign, restricted lateral gaze in both eyes, and staccato speech.

**Figure 1:**
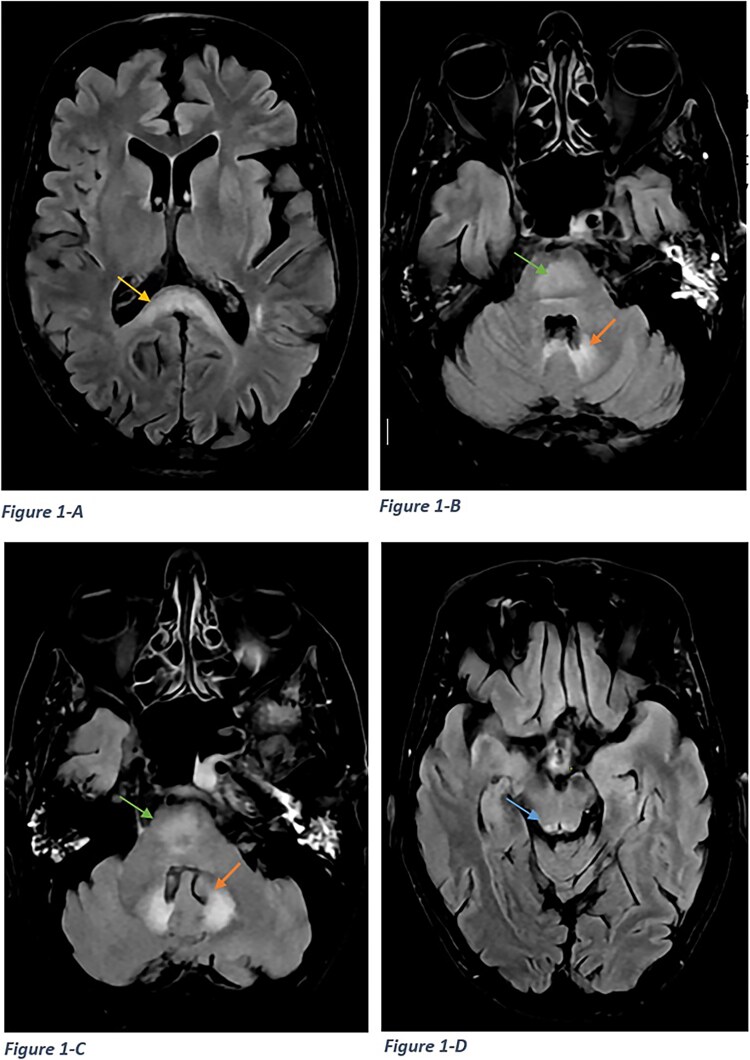
Axial MRI Flair sequence showing high signal in the splenium of the corpus callosum (A), dorsal pons (B-C), bilateral dentate nuclei (C), and tectum (D).

**Figure 2:**
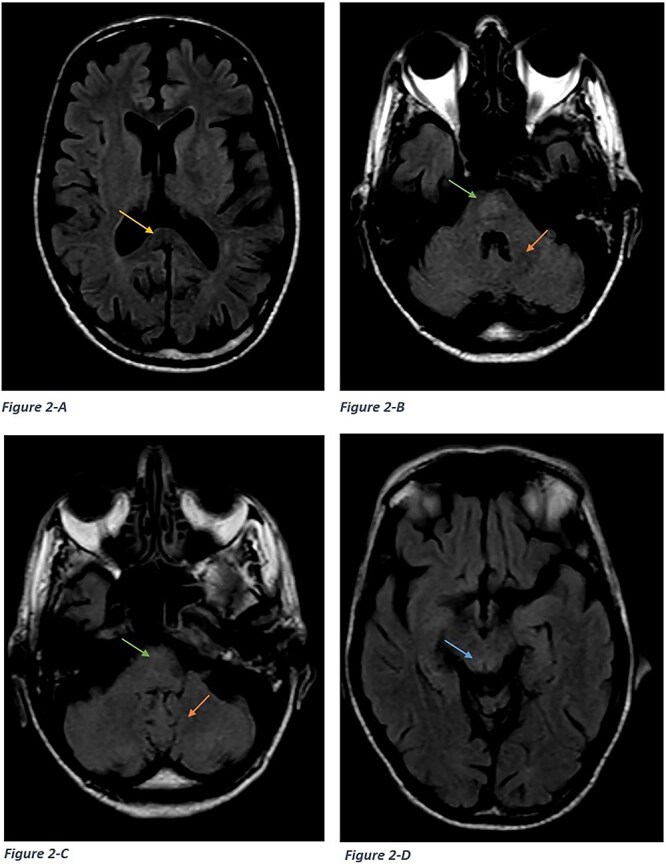
Follow up MRI done five days after cessation of the drug showed significant resolution of the lesions in the splenium of the corpus callosum (a yellow arrow), dorsal pons (B-C), bilateral dentate nuclei (C), and tectum (D).

**Figure 3:**
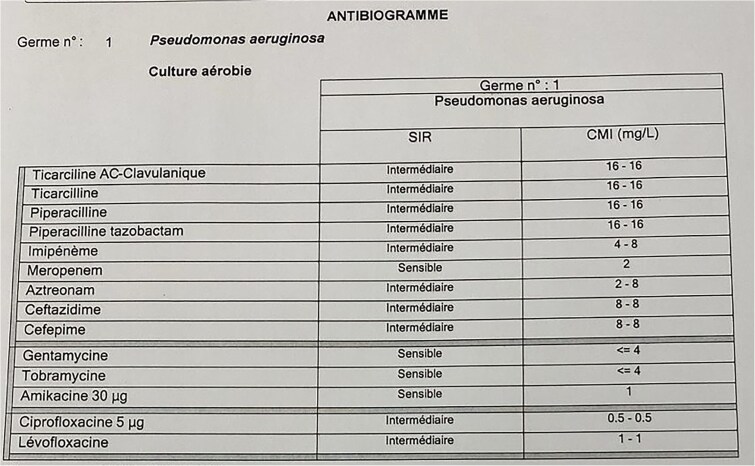
Antibiogram of *Pseudomonas aeruginosa* isolated from the patient’s skull base osteomyelitis. The table displays the susceptibility of the isolated strain to various antibiotics, indicating sensitivity to meropenem, gentamicin, tobramycin, and amikacin.

The patient underwent brain MRI in the Radiology Department, which revealed symmetrical hyperintensities in the bilateral dentate nuclei and tectum on T2-weighted and FLAIR sequences (Figs 1–3). A diagnosis of metronidazole-induced neurotoxicity was suspected, leading to the discontinuation of the treatment. A follow-up MRI performed five days after discontinuing metronidazole showed significant resolution of the lesions (Fig. 2). Clinically, the patient also demonstrated marked improvement.

An antibiogram indicated sensitivity of *P. aeruginosa* to meropenem. The patient was subsequently started on meropenem to complete the treatment for skull base osteomyelitis.

## Discussion

Metronidazole, a widely utilized antiparasitic and antibacterial agent, is among the most commonly prescribed medications worldwide [1]. Skull base osteomyelitis, a severe condition characterized by a heightened risk of complications, including neuroinfection, typically necessitates long-term intravenous broad-spectrum antibiotic therapy. This treatment regimen often involves the administration of ciprofloxacin, ceftriaxone, and metronidazole [2]. However, excessive doses or inappropriate use of metronidazole can lead to the sudden onset of neurological issues such as ataxia, dysarthria, cerebellar signs, or encephalopathy. These symptoms typically resolve rapidly following discontinuation of metronidazole. Patients undergoing metronidazole therapy for conditions such as inflammatory bowel disease, osteomyelitis, or large, undrained abscesses are at an elevated risk of neurotoxicity due to prolonged exposure [2]. Nonetheless, the duration of exposure to metronidazole prior to the onset of symptoms may vary among individuals.

Metronidazole is extensively employed for treating various infections, with certain therapeutic scenarios necessitating prolonged usage [3]. Despite its widespread use, metronidazole typically elicits few adverse reactions, most commonly manifesting as nausea, dry mouth, vomiting, or diarrhea. While neurotoxicity is rare, it can encompass symptoms such as peripheral neuropathy, headache, dizziness, syncope, vertigo, and confusion [2]. Notably, cerebellar toxicity is an uncommon occurrence associated with metronidazole therapy [4]. The onset of cerebellar symptoms following metronidazole treatment varies, with cumulative doses ranging from 25 g to 110 g [5]. Pharmacological studies indicate that the risk of neurotoxicity escalates with doses exceeding 42 g [2].

Ahmed et al. were the first to describe the imaging manifestations of metronidazole toxicity, identifying symmetric abnormal signal patterns within the supratentorial white matter, including the corpus callosum, and within the cerebellum, encompassing the cerebellar deep gray matter nuclei [2, 6]. The proposed mechanism behind these MRI changes involves axonal swelling accompanied by increased water content, resulting in T2 prolongation and ultimately leading to the rapid reversibility of the observed changes upon discontinuation of the drug [6]. Neurotoxicity attributed to metronidazole is believed to occur through various mechanisms, including binding to RNA, DNA, and inhibitory neurotransmitters, as well as inducing both vasogenic and cytotoxic edema [2, 7]. Additionally, genetic predisposition may also contribute to the development of metronidazole-induced neurotoxicity [8].

MRI plays a crucial role in both diagnosing and monitoring these cases, with heightened signal intensity typically observed on T2W/FLAIR sequences [9], consistent with our patient’s findings. Furthermore, MRI may offer prognostic value by predicting symptomatic improvement. In metronidazole-induced encephalopathy, [4] lesions commonly manifest in specific brain regions such as the cerebellar dentate nuclei, tectum, red nucleus, and tegmentum surrounding the periaqueductal gray matter, dorsal pons, medulla, and splenium of the corpus callosum, exhibiting bilateral symmetry [2, 7]. Uncommonly affected areas include the inferior olivary nucleus and the white matter of the cerebral hemispheres [10]. Signal intensity alterations observed on diffusion-weighted images typically signify interstitial edema, except in cases involving the splenium of the corpus callosum, where cytotoxic edema may be present [7]. In our patient, lesions were localized to the splenium of the corpus callosum, dorsal pons, bilateral dentate nuclei, and tectum.

The differential diagnosis of bilateral symmetric T2 hyperintense lesions in the dentate nuclei includes conditions such as methyl bromide intoxication, maple syrup urine disease, enteroviral encephalomyelitis, and Wernicke’s encephalopathy [9]. These conditions must be carefully considered in conjunction with clinical history and imaging findings to establish the correct diagnosis.

Discontinuation of metronidazole treatment typically leads to resolution of both imaging abnormalities and clinical symptoms [6]. Therefore, the use of metronidazole should be approached cautiously, with clear indications, particularly during prolonged courses and when prescribed in higher doses.Haut du formulaire.

## Conclusion

Vigilant monitoring of patients receiving high-dose metronidazole therapy is essential to detect and manage neurotoxicity. MRI serves as an indispensable diagnostic tool for identifying metronidazole-induced neurotoxicity, highlighting the importance of cautious and judicious use of this medication.
